# Gene Therapy Approaches for Atherosclerosis Focusing on Targeting Lipid Metabolism and Inflammation

**DOI:** 10.3390/ijms26146950

**Published:** 2025-07-19

**Authors:** Evgeny Bezsonov, Nikita Chernyi, Mane Saruhanyan, Dariia Shimchenko, Nikolai Bondar, Darina Gavrilova, Mirza S. Baig, Alexander Malogolovkin

**Affiliations:** 1 Laboratory of Molecular Virology, First Moscow State Medical University (Sechenov University), Moscow 119435, Russiamanesaruhanan954@gmail.com (M.S.);; 2 Department of Biology and General Genetics, First Moscow State Medical University (Sechenov University), Moscow 105043, Russia; 3 Department of Biosciences and Biomedical Engineering (BSBE), Indian Institute of Technology Indore (IITI), Simrol, Indore 453552, India

**Keywords:** gene therapy, atherosclerosis, viral vectors, AAV, LDL, LDLR, PCSK9, ncRNA

## Abstract

Atherosclerosis is a complex disease characterized by pathological thickening of the arterial intima. The mechanisms underlying the induction and progression of atherosclerosis are convoluted and remain under active investigation, with key components such as lipid accumulation and local inflammation being identified. Several risk factors (e.g., metabolic disorders, genetic background, diet, infections) have been shown to exacerbate disease progression, but their roles as clinically relevant markers remain to be established. Despite the growing body of evidence on the molecular pathogenesis of atherosclerosis, there is no effective preventive treatment against the development of this disease. In this review, we focus on gene targets for gene therapy as a novel potential approach to cure and prevent atherosclerosis. We critically review recent research demonstrating the therapeutic potential of viral vector-based (adeno-associated virus (AAV) and lentivirus) gene therapy for the treatment of atherosclerosis. We also summarize alternative gene targets and approaches (e.g., non-coding RNA (ncRNA), micro RNA (miRNA), small interfering RNA (siRNA), antisense oligonucleotide (ASO), CRISPR/Cas9) that aim to limit disease progression. We highlight the importance of local inflammation in the pathogenesis of atherosclerosis and propose gene targets with anti-inflammatory activity to inhibit the pathological inflammatory response. In addition, we provide perspectives on the future development of gene therapeutics and their potential applications. We anticipate that recent advances in gene therapy will help to identify new and effective targets to prevent atherosclerosis.

## 1. Introduction

Cardiovascular disease (CVD) is one of the leading causes of mortality in the world, with atherosclerosis being one of the main causes of CVD. It develops in all populations, ages, and sexes, especially at asymptomatic levels (and to be more precise, CVD affected more than 400 million people in the world by 2020) [1]. Atherosclerosis is a complex disease related to pathological thickening of the arterial intima leading to the formation of plaques [2]. The growth of atherosclerotic plaque leads to the decrease of the lumen of a vessel which can impair blood supply to tissues and organs, and change hemodynamics, but also, there is a chance of rupture of advanced plaques leading to the formation of a thrombus with up to complete blockage of blood supply through the affected artery [2]. There are two main components classically associated with atherosclerosis pathogenicity: accumulation of lipids and changes in lipids and lipoproteins metabolism [3], and focal inflammation [4]. These components seem to be intertwined but there are some other factors potentially contributing to atherosclerosis development such as infections [5,6], mitochondrial mutations and dysfunctions [7,8,9], nitric oxide [10], diet [11,12,13]. Changes in hemodynamics play an important role in the development of atherosclerosis via fluid-generated wall shear stress, leading to endothelial dysfunction [14]. Recent studies have also shown that ATP released by red blood cells may significantly contribute to the initiation and progression of atherosclerosis. Well-known risk factors for the development of atherosclerosis include diabetes and hypertension [15]. Diabetes can lead to the development of hyperlipidemia and atherosclerosis due to increased synthesis of very low-density lipoproteins (VLDL) in the liver. Hypertension, in turn, contributes to the development of atherosclerosis through vascular endothelial dysfunction and excessive activation of the renin-angiotensin-aldosterone system (RAAS) and the sympathetic nervous system (SNS) [15] Amyloid deposits (which can be found in different life forms from yeast [16,17] to mammals [18] could be one of the under investigated topics in the context of its potential effects on atherosclerosis development [19].

**Figure 1:**
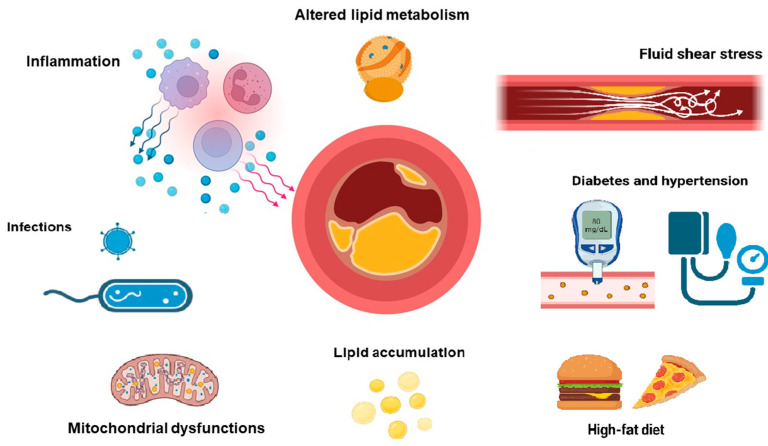
The main pathological factors associated with atherosclerosis development. Accumulation of lipids and changes in their metabolism, together with local inflammation, seem to be one of the major factors contributing to atherosclerosis development. Viral and bacterial infections could also provoke atherosclerosis development. The role of diet and microbiome changes in atherosclerosis development is established, but not well studied yet. High-fat diet is well used to induce atherosclerosis in animal models, along with trimethylamine-N-oxide (TMAO), an atherosclerosis-promoting compound synthesized from a precursor produced by intestinal microbiota. Fluid-generated wall shear stress also leads to the development of atherosclerosis due to endothelial dysfunction. Diabetes and hypertension are significant factors in the development of atherosclerosis.

Despite active clinical use of statins and proprotein convertase subtilisin/kexin type 9 (PCSK9) inhibitors, unfortunately, up to now, there are no effective drugs or treatments completely preventing or reversing atherosclerosis development, which proves the absence of a clear understanding of the pathogenesis of this disease. One of the ways to resolve this issue could be the search for the most promising gene targets leading to the curing of atherosclerosis from the published literature (we have used the PubMed database), which have not been tested yet in clinical trials. The list of these promising targets would help the researchers to choose the next gene target/s for atherosclerosis treatment. Below, we summarize the most promising targets for atherosclerosis therapy/prevention using a gene therapy approach. The main pathological factors contributing to atherosclerosis development are shown in Figure 1.

### 1.1. LDL Role in Atherosclerosis Pathogenicity

Gene therapy aims to treat diseases by introducing therapeutic genes into patients’ somatic cells. The target diseases are either multigenic or single-gene defects in origin. Atherosclerosis is a complex disease that develops over a long period of time, affects multiple cell types in the vessel wall, and involves many different genes working in conjunction with these disease pathways, such as pathways of lipid metabolism, for example. Atherogenesis is estimated to begin as early as the first decade of life, but does not become clinically apparent until decades later [20].

One of the most closely associated with atherosclerosis development classes of molecules is low-density lipoproteins. Low-density lipoproteins are a class of complex units consisting of proteins and lipids, the connection between which is carried out through hydrophobic and electrostatic interactions. Their main function is to transport cholesterol and triglycerides through the bloodstream, thus implementing lipid metabolism. Lipoproteins are classified by size, density, function, relative content of cholesterol, triglycerides and proteins into five large classes [21] (Table 1).

Small, dense LDL particles are particularly rich in cholesterol esters and play a major role in the pathogenesis of conditions such as hypertriglyceridemia and insulin resistance. Foam cell formation is an early event in atherosclerosis. Increased expression of cytokines (such as IL-1β and TNF-α) [22]), chemokines (such as MCP1/CCL2 [22]), and adhesion molecules leads to increased adhesion of monocyte-macrophages to the endothelium and their transmigration into the subendothelial space. There, macrophages and, to some extent, smooth muscle cells (SMCs) accumulate lipids via scavenger receptors, converting these cells into lipid-laden foam cells. Oxidation of LDL by lipoxygenases and other mechanisms (for example, by reactive oxygen species) allows its recognition by scavenger receptors, which do not recognize native LDL, and mediates unregulated cholesterol uptake into cells. SMCs migrated from the media to the intima, proliferate and produce extracellular matrix in the intima, resulting in the formation of atherosclerotic lesions with a necrotic lipid core and a fibrous cap of iSMCs and extracellular matrix. In the worst case, the rupture of these lesions leads to thrombosis and infarction [20].

Many auxiliary risk factors contribute to the development of atherosclerosis and its related complications [23]. Among them, high plasma LDL and low high-density lipoprotein (HDL) levels are key factors promoting atherogenesis. There are also several hereditary diseases, such as familial hypercholesterolemia (FH), that lead to accelerated atherosclerosis and are difficult to influence through lifestyle and dietary changes. Current treatment strategies aim to lower cholesterol levels and reduce the incidence of hypertension and thrombosis through drug therapy. However, these approaches are not effective in all patients in practice [23]. The key elements of successful gene therapy are the identification of suitable target genes that can influence the development and progression of the disease, the efficient delivery of transgenes to target tissues, achieving sufficient gene expression levels for therapeutic effects, and achieving these goals with minimal side effects. In addition, regulated and tissue-specific gene expression is the goal of future gene therapy applications.

### 1.2. LDL Metabolism and Functions

Before selecting potential gene therapy targets and reviewing ongoing research in this area, a brief overview of the main metabolites involved in lipid metabolism processes involved in the development of atherosclerosis pathogenesis is required.

**Figure 2:**
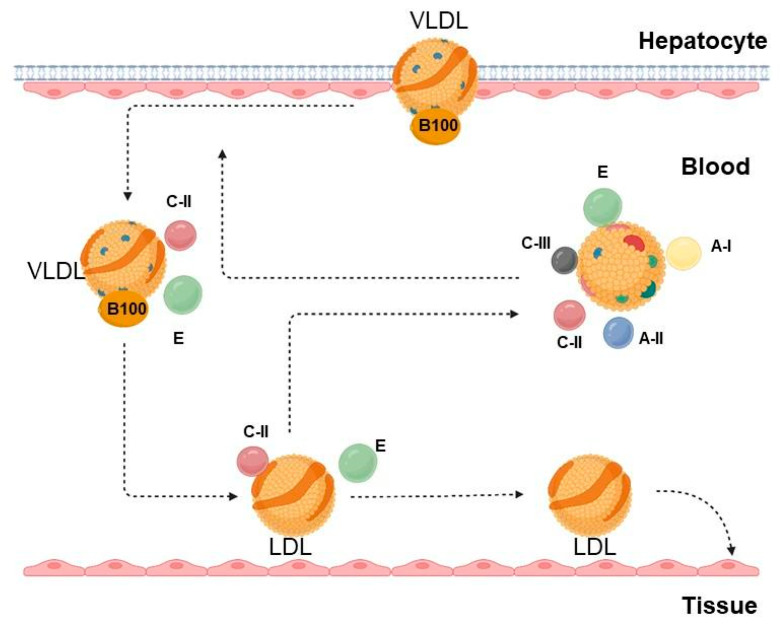
Major pathways of low-density lipoprotein metabolism. LDL—low-density lipoproteins, VLDL—very low-density lipoproteins, LDLs—intermediate-density lipoproteins, HDL—high-density lipoproteins, LPL—lipoprotein lipase, B-100, E, C-II, C-III A-I, A-II—apolipoproteins (adapted from Egorova et al., 2024; Lent-Schochet D, 2024 [21,24]).

Briefly, VLDL synthesis includes the following main steps:

1. Synthesis in the liver (using excess triglycerides derived from free carbohydrates, plasma fatty acids, and chylomicron remnants). VLDL synthesis increases with an increase in intrahepatic free fatty acids, such as with high-fat diets and when excess adipose tissue releases free fatty acids directly into the bloodstream (e.g., obesity, diabetes) [21].
2. As a part of the lipid core of VLDL with the integral protein B-100, they enter the liver capillaries. There, apolipoproteins C II and E are transferred from high-density lipoproteins to VLDL. Apolipoprotein C II, located on the surface of VLDL, activates endothelial lipoprotein lipase, which breaks down triglycerides into free fatty acids and glycerol, which are absorbed by cells.
3. The interaction of VLDL with lipoprotein lipase in tissue capillaries leads to the formation of residual cholesterol-rich VLDL. In this case, their size decreases several-fold and their density grows.
4. Getting into the liver through apolipoprotein E and B-100 receptors, VLDL are either destroyed (approximately half of the particles) or, under the action of liver lipase, are converted into LDL, while apolipoproteins C II and E return to HDL, and only apolipoprotein B 100 remains on LDL. LDL contains ¾ of all plasma cholesterol, and the diameter of the particles decreases again. Their main function is to deliver cholesterol to the cells of the adrenal glands, skeletal muscles, lymphocytes, gonads and kidneys (Figure 2).

LDL:

(1) Are removed from the bloodstream by liver cells via hepatic LDL receptors with the participation of apoprotein B 100 within 2–6 h (40–60% of particles).
(2) The remaining LDL is taken up either by the liver or extrahepatic cells via scavenger receptors (SR). Conversely, with a decrease in fat and cholesterol in the diet, the number of these receptors increases (“up-regulation”). With the entry of chylomicron cholesterol into the liver and an increased content of saturated fats in the diet, the number and binding capacity of hepatic LDL receptors decrease (“down-regulation”). The excess LDL that is not taken up by the liver LDL receptors (LDL-R) is removed from the bloodstream via extrahepatic SR “scavenger receptors” (mainly in macrophages).

Monocytes migrate to the subendothelial space and transform into macrophages. The latter take up more oxidized LDL, turning into foam cells within atherosclerotic plaques [25]. LDL can be modified (in the form of oxidized forms, the amount of which increases with increased levels of reactive oxygen species in the body, in the process of general oxidative stress) and can be recognized by the immune system as unwanted elements. Macrophages then capture them and remove them from the body in the form of HDL. When LDL levels are excessively high, macrophages become overloaded with lipid particles and settle in the artery walls, forming atherosclerotic plaques [21].

## 2. Atherosclerosis Gene Therapies Targeting Lipid and Lipoprotein Metabolism

Hepatocytes are primary cells for gene therapy for the treatment of atherosclerosis due to their central role in lipoprotein metabolism, and another target would be the arterial wall cells, where atherosclerotic lesions develop in the intima [26]. Both viral and non-viral vectors initially demonstrated relatively low efficiency and lack of selectivity in vascular endothelial cells, but vascular cell-targeting vectors have been developed over the course of research [27]. Long-term expression is possible with lentiviral vectors) [28] or adeno-associated viral vectors (AAV) [29,30]. Persistent gene expression has also been achieved in the liver with both vectors [31]. In addition, gene transfer via muscle cell transfection may also be feasible for the production of secreted apolipoproteins [32].

### 2.1. LDL Receptor Targets

An LDL receptor is a cell surface protein responsible for the recognition and endocytosis of LDL, and disruption of the LDL receptor obviously can lead to an increase in serum LDL-C. Thus, one can consider LDL receptors as one of the important targets for gene therapy capable of affecting blood LDL-C levels.

Elevated serum LDL-C levels result from the inability of familial hypercholesterolemia (FH) patients to clear circulating cholesterol caused by mutations in any of the major genes involved in LDL uptake, including LDL receptor [33], apolipoprotein B [34], proprotein convertase subtilisin/kexin type 9 (*PCSK9*) [35], and low-density lipoprotein receptor adaptor protein 1 (*LDLRAP1*) [36]. Among these genes, LDL receptor mutations are responsible for 80–90% of hypercholesterolemia cases [37,38]. Elevated plasma LDL-C concentration is a significant correlate of atherosclerosis risk [39]

Elevated plasma LDL-C (low-density lipoprotein cholesterol) levels can arise from a combination of environmental influences and genetic factors. However, certain monogenic disorders have also been linked to significantly increased LDL-C concentrations [40]. Among these, familial hypercholesterolemia (FH) is a well-known genetic condition characterized by extremely high cholesterol levels and a heightened risk of severe cardiovascular disease. FH primarily results from mutations in key genes responsible for LDL-C uptake, impairing the liver’s ability to clear LDL from the bloodstream. This leads to persistently high plasma LDL-C levels and accelerates the development of premature atherosclerosis and related complications [40]. A potential therapeutic approach involves liver-targeted gene therapy to introduce functional LDL or VLDL (very-low-density lipoprotein) receptors, partially restoring hepatic LDL uptake and lowering circulating cholesterol levels. As shown in animal models of FH, after 8 weeks of high-fat diet, the experimental group experienced a decrease in cholesterol levels (on average 4.5 mM/L vs. 6 mM/L in the control group) after *LDLR* gene transfer and protection from atherosclerosis after VLDLR gene transfer [26]. Mice with a deletion of the LDL-R receptor were used as a model of FH. LDLR mRNA was encapsulated in exosomes for forced expression of *LDLR* [26].

AAV8-based vector was used, and robust hepatic *LDLR* expression was driven by the thyroxine-binding globulin promoter (TBG.mLDLR). Based on these advances, more than two decades after the first HoFH gene therapy clinical trial, a new phase I/IIa human clinical trial testing AAV8. TBG.hLDLR (NCT02651675) was initiated in March 2016 (NCT02651675), which was terminated in 2020 due to financial reasons. Ex vivo transplantation of transduced autologous hepatocytes expressing *LDLR* also resulted in a modest reduction (from 737 mg dL^−1^ to 595 mg dL^−1^) in cholesterol levels in some patients [41]. It is worth noting that this treatment strategy may face challenges in translating to the pediatric population. Childhood livers undergo significant growth, which is important because rAAV vector DNA is stored primarily as episomes (extrachromosomal DNA) and is not replicated during cell division. Thus, rAAV-based gene addition treatments given in childhood are likely to have only a temporary effect [42].

Elevated levels of cholesterol are observed in FH disease, which is caused primarily by mutations in the LDL receptor gene [43]. One of the developed approaches is to translate a functional receptor to recover its expression via mRNA delivery by exosomes—nanoscale intercellular transporters of biological macromolecules. Exosomes are exploited for target delivery of nucleic acids, proteins and drugs to specific organs and tissues. Another advantage of exosome-based therapy is low toxicity and high biocompatibility. Besides that, exosomes are easy to design [44]. The effectiveness of *LDLR* mRNA exosomes in the reduction of serum cholesterol level and preventing progression of atherosclerosis had been examined on the *LDLR*−/− FH murine model. *LDLR*−/− mice were fed a high-fat diet for 2 months, subsequently they were administered with exosomes (4 mg/g weekly during 8 weeks) containing *LDLR* mRNA and empty control exosomes. In mice treated with Exo LDLR, the area affected by atherosclerotic plaques of the aortic arch decreased by about 3 times compared to controls with PBS and an empty exosome. Total cholesterol level and LDL-C in Exo LDLR mice were reduced twofold. As for the inflammatory state, after Exo LDLR treatment, macrophage infiltration was also decreased: CD68+ area occupies approximately 2–3% of the aortic area, which is about 6 times less than the average area of CD68+ of the PBS control group [26].

Since mutations in the gene encoding the LDL receptor are implicated in the pathogenesis of FH and are the main cause of disease development in most cases, this gene has become a target for correction using CRISPR/Cas9 [45]. A study involving somatic cell *LDLR* gene correction via CRISPR was conducted with the aim of determining the efficiency of CRISPR/Cas9-mediated *LDLR* gene editing delivered by AAV in preventing FH and atherosclerosis in the LDLR E208X murine model [46]. The AAV8 CRISPR Cas9 system had been constructed, which was used for gene editing in LDLR E208X mice at the neonatal stage [46]. The neonatal mice were divided into four groups according to received treatment: 1–LDLR E209X + 5 × 10^10^ genome copies of AAV-CAS9, 2–LDLR E208X + 5 × 10^11^ genome copies of AAV-gRNA-Donor, 3–LDLR E208X + 5 × 10^10^ genome copies of AAV-CAS9 + 5 × 10^11^ genome copies AAV-gRNA donor sequence, 4–wild type + PBS, by subcutaneous injection, and further placed on a high fat diet. The data presented in their experiment has indicated partial expression of LDLR mRNA in group 3, amounting to only 11% of wild-type mice; the blood Ldlr level was 18% of the wild type. The perimeter of the aortic arch occupied with atherosclerotic lesions in groups 1 and 2 was approximately 90–95%, and in 3rd there was a 2-fold decrease compared with 1 and 2. The average value of the total blood cholesterol level (mmol/L) in group 3 is reduced by 2 times compared to group 1 [46]. In the given preclinical study, the highest tolerable dose of AAV—5 × 10^11^ genome copies. AAV8 showed high hepatocyte transduction, but off-target transduction could not be excluded as well. Although AAV serotypes often have high tropism for the desired tissue or organ, weak transduction of non-targeted tissues/organs can still occur. The potential off-target effects of CRISPR Cas9 genome editing also require further research.

Thus, *LDLR* could serve as one of the most prospective targets of gene therapy of atherosclerosis with different options related to the delivery of more copies of this gene and/or gene editing in case of need for the correction of mutations affecting blood cholesterol levels.

### 2.2. Targeting Apolipoproteins

A high level of ApoB expression is often associated with increased risk of atherosclerosis [47]. In order to prevent the development of pathology, it is possible to ablate ApoB expression. RNA interference-based technologies are powerful tools for such therapy. The usage of short hairpin RNA (shRNA) is an effective technique for gene silencing via RNA interference.

A study to estimate AAV-mediated short hairpin RNA delivery for ApoB silencing in mice had been conducted [48]. C57BL/6 mice were injected intravenously with scAAV serotype 8 expressing shApoB (AAV-shA10) to inhibit ApoB. Data in their experiment indicate that liver Apob mRNA expression was abridged with increasing dose from 10^8^ to 10^11^ genome copies of scAAV8-shA10, up to 95% compared to PBS control. Cholesterol level reduction was reached up to 79% comparable with PBS control. It is stated that a dosage no more than 10^10^ genome copies does not demonstrate toxic effects on the liver [48].

High plasma levels of lipoprotein (a) are considered to exacerbate the risk of atherosclerosis [49], although molecular mechanisms are still unclear. Genetic studies demonstrated that there is a causal link between genetic polymorphisms in the *LPA* gene and Lp (a) concentration and, as a consequence, coronary heart disease risk [50]. Previous studies indicate that rare variants of *SCRAB1*, the gene encoding Scavenger receptor class B type 1, are linked to coronary heart disease development in humans [51]. However, it was found that there is no association of *SCRAB1* mutations with coronary artery disease [52]. Based on this contradictory data, it is not yet possible to draw an unambiguous conclusion about whether it is necessary to apply gene editing techniques if such a mutation is detected in patients.

Ligand-conjugated antisense oligonucleotide (ASO) AKCEA-APO (a)-LRx (pelacarsen) was developed for apo (a) inhibition. In a phase 2b double blind placebo controlled trial, 286 patients with Lp (a) levels 60 mg/dL or above took part. The participants received pelacarsen at doses of 20 mg, 40 mg and 60 mg every 4 weeks, 20 mg weekly or every 14 days. Pelacarsen administration has resulted in a decrease of Lp (a) levels by 35% at 20 mg every 4 weeks, 56% at 40 mg and 58% at 20 mg every 2 weeks, whereas placebo reached only 6% [53].

Apolipoprotein E (ApoE) functions as a ligand for lipoprotein clearance (a metabolic process in which lipoprotein particles are removed from the blood by receptor-mediated endocytosis) and plays an important role in the metabolism of triglyceride-rich VLDL, as well as in residue metabolism, in addition to exhibiting anti-inflammatory and antioxidant properties [54]. Thus, *ApoE* gene transfer can be used to treat hyperlipoproteinemia type III, which is caused by the presence of specific *ApoE* isoforms. It has been shown that *ApoE* expression in macrophages reduces the area of atherosclerotic lesions: in *ApoE*−/− mice from the experimental group, after transplantation of bone marrow transduced with a vector expressing human *ApoE* at the age of 5 weeks and sacrificed at the age of 13 weeks, the size of atherosclerotic plaques was 4847 ± 2410 μm^2^ ± SEM, and in mice from the control group—12,837 ± 7166 μm^2^ ± SEM) without significant changes in the level of cholesterol in the blood plasma. Moreover, it is worth noting that this works precisely at the early, but not at the late stages of atherogenesis—with a similar transplantation at the age of 10 weeks and sacrifice at 26 weeks, no significant differences in the size of atherosclerotic lesions were recorded [55]. However, despite all the facts about the important role of apolipoprotein E in the development of atherosclerosis, as well as the relatively small size of the gene (3.7 kb), which allows the use of the widely available AAV vector as a therapeutic gene delivery system, very few studies have been published in this direction to date. The effects of Lv-hEp-GFP lentiviruses in vivo were studied by Xu Y and colleagues by administering intravenously to 9-month-old *ApoE* −/− mice to express a human ApoE mimetic peptide named hEp [56]. The mice were kept for 18 weeks on a chow diet. In comparison to the control group transfected with Lv-GFP lentivirus (Lv-GFP), injection of Lv-hEp-GFP lentivirus achieved the expression and secretion of hEp in the *ApoE*−/− mice liver. The expression of hEp was observed to reduce the atherosclerotic lesions in the aorta by 24.2% (the experimental group showed a decrease in atherosclerotic lesions by 0.94 ± 0.06 mm^2^ versus 0.81 ± 0.07 mm^2^ in the control group) [56].

It is also worth mentioning that the hepatic lipase gene may be an interesting target for gene therapy due to the important role of this enzyme in the breakdown of triglyceride-rich lipids (VLDL and chylomicrons) [57]. Patients with hepatic lipase deficiency exhibit a variable phenotype that may include hypertriglyceridemia with triglyceride-enriched LDL and HDL particles, hypercholesterolemia, VLDL and, in some cases, premature coronary heart disease [58]. In view of these properties, gene transfer of the hepatic lipase into mice deficient in the corresponding gene could help to normalize the lipid profile. A similar study was performed, however, in the context of *GPIHBP1* deficiency [59].

### 2.3. Proprotein Convertase Subtilisin/Kexin Type 9 Gene Therapy Target

Pcsk9 is an enzyme secreted by hepatocytes. Its function includes regulation of Apolipoprotein B secretion, ApoB elimination from plasma, promotion of lysosomal degradation of the receptor for low-density lipoprotein particles, thus preventing accumulation of excess cholesterol [60]. It is known that gaining the Pcsk9 function mutations is coupled with cardiovascular diseases and autosomal dominant hypercholesterolemia, while their loss lead to decreased LDL-C levels in plasma, consequently mitigating the risks of cardiovascular diseases [61]. Different approaches are applied for Pcsk9 inhibition in order to lower blood cholesterol levels or prevent coronary heart disease.

A promising approach to gene therapy for atherosclerosis is also the inactivation of the secreted enzyme Pcsk9, which destroys lipoprotein receptors [35]. In the event of hyperactivity of the enzyme, a number of receptors will be affected, resulting in an excess of LDL in the blood. To prevent this from happening, drugs that bind to the Pcsk9 enzyme and reduce its activity are used in the clinic, for example, alirocumab and evolocumab [62]. Two studies (FOURIER and ODYSSEY Outcomes study) showed a significant reduction of cardiovascular disease risk relative to statin therapy (15% over 2.2 and 2.8 years of follow-up, respectively) [62,63]. However, regular administration of the drug is required to maintain the effect. Aside from the monoclonal antibodies mediated target protein neutralization, the use of the messenger RNA (mRNA) encoding the corresponding protein is possible. The two main classes of the latter drugs are based on siRNAs and ASOs. siRNAs are mRNA molecules composed of two strands, including the guide strand that consists of a complementary fragment to the target mRNA sequence. When binding to the complementary mRNA fragment, the RNA-induced silencing complex is assembled, which causes cleavage of the target mRNA. It is important to note that the guide strand remains uncleaved; subsequently, a single fragment possesses the ability to assist in degrading multiple mRNAs, which is likely to contribute to prolonging the effects of siRNA therapy [64]. ASOs, in contrast, are single-stranded modified DNA molecules. As they complementarily bind to the target mRNA, its functions are inhibited through various mechanisms, among which the RNase H1 cleavage is one of the most important. In such cases, mRNA cleavage occurs in a one-to-one stoichiometry [65].

RNA interference served as a base for the development of Inclisiran (Leqvio^®^), which is a GalNac-conjugated *PCSK9* small RNAi therapy drug that inhibits the synthesis of Pcsk9 by silencing the *PCSK9* gene in hepatocytes and thereby lowers cholesterol levels [66,67]. The siRNA conjugates to the sugar N-acetylgalactosamine (GalNAc), which binds to the ASGPR hepatocyte receptors, mediating cellular uptake as well as liver selectivity. The use of siRNA after the initial two administrations with a three-month interval allows for twice-yearly dosing. Long-term LDL-C reduction offers potential adherence benefits in comparison to antibodies and daily oral lipid-lowering agents such as statins or ezetimibe. Inclisiran has shown the ability to significantly reduce LDL-C levels: with an average baseline LDL-C level of 153 mg per deciliter, the change was 39.7% compared to the placebo group (on the 510th day after the injection of inclisiran sodium in an amount of 300 mg) [68,69]. Overall, 3457 patients with heterozygous familial hypercholesterolemia or atherosclerotic cardiovascular diseases enrolled in 3 randomized, placebo-controlled clinical trials. Among 1561 participants with atherosclerotic cardiovascular disease in study 1, those who received Leviqo (284 mg, subcutaneously, 510 days after injection) had an average reduction of LDL-C by 51% compared with placebo (1%). In the second study, the Leviqo group experienced an average LDL-C decrease of 46% and 4% in the placebo group. The three studies have displayed an average decrease of LDL-C by 40% for Leviqo and 8% for placebo groups, respectively. Deaths and other serious adverse effects were not identified (FDA). Another therapeutic acting as a Pcsk9 inhibitor, human monoclonal antibody—alirocumab, was approved by the FDA in 2015 for the treatment of heterozygous familial hypercholesterolemia and atherosclerosis.

The ORION-4 study, initiated in 2018 (with a planned completion date of 2049), is aimed at obtaining definitive and quantitative data on the impact of intracellular PCSK9 inhibition on cardiovascular events, tolerability, and safety (NCT03705234) [70]. Ding et al. presented the first research of *PCSK9*-targeted gene therapy in mice [71], in which an adenoviral vector expressing a gRNA targeting exon 1 of mouse *PCSK9* and Cas9 was engineered. Three to four days after administration of the adipocyte-expressing adenovirus vector, ~50% mutagenesis was detected at the target site of *PCSK9* (exon 1), resulting in a significant reduction in *PCSK9* expression levels and a 35–40% reduction in total cholesterol (101 mg/dL with CRISPR-*PCSK9* virus vs. 157 mg/dL with GFP virus (*p* = 0.0079) and vs. 161 mg/dL without virus (*p* = 0.0079), N = 5 per group), with no adverse effects observed. This method is constrained by the need to use an adenovirus-based vector, prone to an immune response; thus, it does not appear to be optimal for use in humans [72]. Adeno-associated virus was used instead of adenovirus, with a Cas9 ortholog from *Staphylococcus aureus* (SaCas9) in the attempt to edit *PCSK9* [73]. Since this ortholog is almost 1 kb smaller than SpCas9, this gene can be placed in an adeno-associated viral vector together with gRNA targeting the *PCSK9* gene [73]. Hepatocyte-tropic AAV serotype 8 with the SaCas9 expression cassette driven by the thyroxine-binding globulin (TBG) promoter was used. One week after injection (2 × 10^11^ copies of the viral genome), C57BL/6 mice showed the appearance of PCSK9 deletions of more than 40% at any locus in the whole liver tissue, and PCSK9 protein and total cholesterol levels were significantly reduced compared to baseline by 95% and 40%, respectively [73].

Structural reorganization of the I-CreI DNA recognition surface was used to develop a first-generation meganuclease, named M1PCSK9, that recognizes a DNA sequence in exon 7 of the human and macaque *PCSK9* gene. AAV serotype 8 viral vector containing the liver thyroxine-binding globulin promoter for expression (i.e., AAV8-M1PCSK9) to edit primate *PCSK9* in vivo was utilized. 6 × 10^12^ copies of the viral genome were injected into the livers of rhesus macaques [74]. As a result, the authors reported that circulating Pcsk9 levels in macaque liver cells were decreased by 59% and LDL levels by 34% compared to baseline [74]. Meganucleases did not edit all *PCSK9* genes in the cells: genetic analysis showed that 40–65% of the target gene fragment (% of indel) was mutated, this was enough to reduce low-density lipoprotein levels [74]. However, the publication did not provide the initial measured values for Pcsk9 and LDL levels, which makes it difficult to objectively evaluate the results. Although the use of meganuclease is advantageous as it allows the AAV instead of AV usage, the main observed drawbacks were the possible off-target editing and immune response. The use of lipid nanoparticle delivery systems for CRISPR/Cas gene editing tools may help to bypass these challenges. Such nanoparticles have been employed with a guide RNA (gRNA) designed to target a splice site of PCSK9 mRNA in non-human primates [72]. Donor and acceptor splice sites are characterized by GT and AG sequences, respectively, and nucleotide exchanges at these sites can disrupt the normal splicing of precursor mRNA.

AAV-mediated CRISPR Cas9 delivery was used to target the *PCSK9* gene [75]. AAV-CRISPR system was constructed with self-cleaving AAV vector, which allows to limit *CAS9* expression, thus reducing adverse immune response to genome editing, SaCas9, chimeric sgRNA targeting *PCSK9* and the flanking sites of gRNA. 2 × 10^11^ genome copies of AAV8-SaCas9/PCSK and SiAAV8-Sacas9/PCSK V1 were administered via tail injection to C57/BL6J mice. Blood Pcsk9 was reduced by 80% (protein level), and serum cholesterol levels dropped by 35% 24 weeks of treatment [75]. To confirm the absence of toxic effects, histological examination was performed 24 weeks after treatment, and no traces of toxic effects were detected [75].

Adenovirus carrying SpCas9 and a gRNA targeting the sequence encoding human *PCSK9* was administered to murine livers with transplanted human hepatocytes, using a CRISPR control to treat mice with chimeric livers (FRG mice) [76]. It was observed that the levels of Pcsk9 secreted by human hepatocytes in the blood decreased by an average of 52% following treatment compared to baseline, whereas murine Pcsk9 blood levels more than doubled from the initial level. Overall, this study demonstrates encouraging results for further investigations involving human cells, applying not only adenoviruses but also other vectors such as AAVs and AAV3B for targeting human hepatocytes.

Extracellular vesicles (EVs) are powerful tools for gene delivery. EVs are nanoscale membranous particles released by cells and include exosomes, microvesicles and apoptotic bodies. There was a study that aimed to determine whether EVs could be effective for CRISPR/Cas9 RNA protein complex delivery to knockout the Pcsk9 gene in primary murine hepatocytes ex vivo [77]. EVs contained rapamycin-interacting complex FKBP12/FRB and vesicular stomatitis virus protein—FKBP12/FRB/VSV-G EVs. The dosage 5 × 10^11^ particles of Cas9EVs with murine *PCSK9* gRNAs resulted in a reduction of *PCSK9* mRNA and an increase in LDLR levels by 1.8 ± 0.4 times compared to the control group using gRNAs not targeting *PCSK9* (“no target gRNAs”) [77]. Gene inactivation with base editors is more precise and considered to cause no double-strand breaks [78].

Despite promising results, there are challenges for further clinical investigations in humans due to the risk of severe immune responses to viral proteins. Extracellular vesicles have advantages such as general low toxicity and immunogenicity, and the ability to carry cargo to specific cells/tissues due to the presence of receptor-specific proteins and lipids on the membrane [79]. But as agents for gene therapy, EVs face the following limitations: technological difficulties of production and purification, underdevelopment of regulatory mechanisms, safety quality control protocols and procedures, absence of standardized approaches of EV therapies both in vitro and in vivo [80].

In the follow-up study, it was shown that CRISPR base editing with a gRNA targeting human *PCSK9* lowered the levels of human Pcsk9 in plasma as well as the total cholesterol in the *PCSK9* knock-in mouse (liver-specific) model [81]. Authors claim the base editing to be more precise compared with genome editing; moreover, it displays no off-target effects, chromosomal translocations or indels [81].

Lipid nanoparticle mediated delivery of CRISPR adenine editor to induce single nucleotide loss-of-function mutation in *PCSK9* gene in *Macaca fascicularis* was tested. The four primates were treated with 3 mg/kg of the lipid nanoparticle with adenine base editor and gRNA, and the other two monkeys received PBS as a control via intravenous infusion. Pcsk9 reduced by nearly 90% and cholesterol by 60% in monkeys that received treatment from baseline before treatment [82].

A similar approach was applied by another group who investigated lipid nanoparticle carrying mRNA encoding adenine base editor, and gRNA targeting *PCSK9* in cynomolgus macaques and C57BL/6J mice liver as models of heterozygous familial hypercholesterolemia. Base editing has resulted in a disruption of Pcsk9 synthesis and, as a consequence, a reduction of plasma LDL in monkeys. The macaques were infused with 0.75 mg/kg RNA and 1.5 mg/kg RNA single and repeat doses. 58% and 14% LDL-C decrease in mice and monkeys, respectively, was observed. Plasma Pcsk9 was reduced by 95% for mice and 32% for monkeys. No off-target effects were detected [83]. While LNPs show great promise as agents for gene therapy, there are considerations for their further clinical translation. For instance, there are issues with biodistribution—LNPs must reach specific tissue and be taken up by target cells without being eliminated by the mononuclear phagocytic system, renal filtration and other physiological factors for successful in vivo application [84]. Also, the physiological environment can affect LNP stability, and the toxicity of LNPs is not fully investigated. In the research mentioned above, 3 mg/kg seems to be a tolerable dose of LNPs for non-human primates, but for humans, actual amounts still must be clarified.

The encouraging results of the aforementioned studies have given insight into the development of novel and safe drugs. Verve Therapeutics developed a CRISPR-based drug (VERVE-101) targeting Pcsk9 to change single nucleotides of the gene in hepatocytes. It is composed of adenine base editor mRNA and gRNA directing Cas9n to the target *PCSK9* gene, all packaged in a lipid nanoparticle [85]. The Heart-1 clinical trial involved eight male and two female patients with heterozygous familial hypercholesterolemia, confirmed atherosclerotic cardiovascular disease and average LDL cholesterol of 193 mg/dL [86]. Three patients received the drug at a dosage of 0.1 mg/kg, another three received 0.3 mg/kg and 0.45 mg/kg, and one patient received 0.6 mg/kg, single infusion intravenously. It has been reported that Pcsk9 levels in blood was reduced by 47% in individual who received 0.6 mg/kg, two persons who were infused with highest dose experienced Pcsk9 decrease by 59% and 84% and in patients with 0.3 mg/kg reduction by 7%, 24% and 40% for each from baseline. LDL-C levels were reduced by 39% and 48% for those who were treated with 0.45 mg/kg and 55% for 0.6 mg/kg. For patients at a dose of 0.3 mg/kg, a significant decrease in LDL-C was not recorded, amounting to only 9% and 10% from baseline. Results are encouraging, but this is an early clinical trial phase; therefore, some questions may arise, such as the long-term effects of gene editing (more than 6 months) and what the optimal doses for durable efficacy and safety are on larger cohorts.

Current studies demonstrate that gene modulation of dyslipidemia-associated targets (in particular, *PCSK9*) can have a positive effect not only on the atherosclerotic process but also on the risk of atrial fibrillation (AF). This effect can be mediated by several mechanisms:

- Reduction in systemic inflammation—improvement of the lipid profile leads to a decrease in atherosclerotic vascular lesions and the accompanying inflammatory response.
- Improvement of endothelial function—normalization of vascular function can reduce atrial ischemia and electrical instability.
- Direct effect on cardiomyocytes—some studies indicate a possible direct antiarrhythmic effect of Pcsk9 inhibitors.

In patients with AF and significant coronary artery disease, a comprehensive management strategy is especially important, including both optimal antithrombotic therapy and control of cardiovascular risk factors [87]. The authors emphasize the need for a personalized approach to such patients, which opens up opportunities for including gene therapy in treatment algorithms.

A promising direction seems to be a combination of gene therapy aimed at correcting dyslipidemia with modern anticoagulants and antiarrhythmic drugs. However, as noted in the review, additional research is required to determine optimal strategies for managing this complex category of patients.

Thus, *PCSK9* is another example of a promising gene therapeutic target related to editing of the genome for the reduction of expression levels/activity of Pcsk9.

### 2.4. Angiopoietin-like 3

Angiopoietin-like 3 (Angptl3) is a liver protein that inhibits the enzymes lipoprotein lipase and endothelial lipase and is a regulator of lipid and lipoprotein metabolism [88]. It is reported that in patients who carry complete loss of Angptl3 function variants, plasma LDL cholesterol and triglycerides are profoundly reduced, as well as the risk of cardiovascular disease development is lowered [89]. Loss-of-function mutations in the gene encoding apolipoprotein C3 also reduce the risk of coronary cardiovascular disease by 40% [90,91]. In 2017, in 3 patients with full *ANGPTL3* loss of function mutations, no atherosclerotic plaques were detected [92]. It may raise a question whether *ANGPTL3* is essential and whether its knockout poses a threat to life. Almost complete knockout of the *ANGPTL3* gene in C57BL/6 mice after administration of AAV9 had been achieved with no serious adverse and toxic effects being reported [78]. Deletion of the *ANGPTL3* gene in the Huh7 cell line also did not lead to cell death [93]. Although *ANGPTL3* knockdown disrupts cell proliferation in zebrafish embryos [94], this manipulation does not threaten the adult individual. It was proposed that complete loss of function of *ANGPTL3* does not have a negative impact on human health [95]. A drug that inhibits Angptl3 has been developed and approved. Evinacumab (Evkeeza^®^)—a monoclonal antibody acting as an Angptl3 inhibitor used for patients with HoFH, approved by the FDA and EMA in 2021, has proven to be effective and safe. In the study involving 65 individuals who received Evkeeza weekly during 24 weeks, blood LDL-C levels were decreased by 47% on average. (https://www.ema.europa.eu/en/medicines/human/EPAR/evkeeza (accessed on 5 May 2025)). The most common side effects are inflammation of the nose and throat, flu, dizziness, and anaphylaxis. Based on the data provided, we cannot establish the causal relationship between ablation of *ANGPTL3* gene expression and deterioration of the quality of life up to a lethal outcome.

Lipid nanoparticle formulations have been successfully used for CRISPR/Cas delivery. A bioreducible lipid nanoparticle was created carrying the CRISPR/Cas9 system that allows SpCas9 mRNA and sgRNA delivery to the liver in order to perform knockdown of the *ANGPTL3* gene target locus [95]. The lipid nanoparticle contains a leading tail-branched bioreducible lipidoid (306-O12B) with excipient lipids [95]. Wild-type C57BL/6 mice were administered the lipid nanoparticles encased with cas9 mRNA and *ANGPTL3*-specific gRNA, as well as a control RNA dose of 3 mg/kg. There was reported a stable long-term reduction of serum Angptl3 protein by 65.2%, LDL cholesterol level dropped by 56.8%, TAG reduced by 29.4% whereas MC-3 LNP, which is considered to be a gold standard lipid nanoparticle for mRNA delivery, reduced Angptl3, LDL-C and TAG by 25%, 15.7%, 16.3%. The therapeutic effect was stable for at least 3 months after injection [95]. Authors by computational analysis detected nine sites with high probability of off-target effects for sgRNA targeting *ANGPTL3*, but NGS revealed no undesirable effects [95]. It is unclear whether favorable treatment effects last for years without severe consequences, particularly in humans. Besides, computational methods often omit sgRNA-independent off-target sites) [96], so deeper analysis is required.

Another approach in modulating Angptl3 activity and thus lowering LDL-C and TAG levels is RNA interference therapy. ARO-ANG3 is a siRNA conjugated with N-acetylgalactosamine, a drug degrading *ANGPTL3* mRNA, thus causing silencing of the *ANGPTL3* gene [97]. Single dosage of 100 mg, 200 mg, 300 mg decreased blood Angptl3 by ~58%, ~64%, ~80%, respectively. No life-threatening adverse effects were recorded [98].

*ANGPTL3* is a suitable target for gene therapy not only due to its important role in lipid metabolism, but also due to its localization. Angptl3 is a hepatic enzyme and the liver is considered to be a convenient and attractive target for in vivo gene therapy [98,99].

### 2.5. Apolipoprotein C III (APOC3)

*APOC3* encodes a small glycoprotein (with a mass of 8.8 kDa) that is synthesized in the liver mostly, regulates triglyceride metabolism, and interacts with LDL, HDL and triglyceride-rich lipoproteins [100].

Apolipoprotein C III (apo-CIII) contributes to hypertriglyceridemia through multiple mechanisms. Its primary role involves the inhibition of lipoprotein lipase (LPL), the enzyme that breaks down triglycerides in adipose and muscle tissue capillaries. Additionally, at elevated concentrations, apo-CIII suppresses hepatic lipase (HL), a liver-synthesized enzyme with triacylglycerol lipase and phospholipase-A1 activity. Apo-CIII also promotes the production and release of very-low-density lipoprotein (VLDL) from the liver. Furthermore, it slows triglyceride clearance by disrupting the interaction of apoB and apolipoprotein E (ApoE) with hepatic receptors, impairing their uptake [101].

Several studies revealed that CRISPR inactivation of *APOC3* had an antiatherogenic effect in hamsters and rabbits [96,102]. CRISPR-mediated deletion of the *APOC3* gene in Syrian golden hamsters was carried out [102]. *APOC3*−/− hamsters revealed to have fewer atherosclerotic lesions in thoracic and abdominal arteries after high-fat diet compared to the wild type [103]. *APOC3*-deficient rabbits also displayed reduced atherosclerotic plaque and plasma TAG levels after high-fat diet. On a normal diet, *APOC3* knockout rabbits had 50% TG compared to controls. Off-target effects of CRISPR/Cas9 in hamsters were not investigated, and there was no data on the analysis of the presence of antibodies against Cas9 in animals participating in experiments [96]. Also, the authors did not study biochemical markers of inflammation in the liver or other organs that could appear because of gene editing. The results of preclinical studies are not yet sufficient for translation to humans. Although there are other Cas proteins that appear to be less immunogenic, their long-term safety and efficacy remain unclear [103]. In some clinical trials involving the CRISPR system, editing efficiency was low, and most clinical trials currently are in early phases [104].

Volanesorsen (sold under the brand name Waylivra™ and discovered and developed by the Ionis Pharmaceuticals company) is an ASO designed to block the production of apolipoprotein C III, which decelerates the breakdown of fats, and was authorized in the EU for medical use as Waylivra on 3 May 2019. The phase three clinical trial of Waylivra on 66 familial chylomicronemia syndrome patients showed that in patients exposed to the drug for three months, on average, blood triglyceride levels decreased by 77%, compared to the placebo group, where an increase of 18% was detected [105].

ARO-APOC3 (Plozasiran) is a siRNA therapeutic that inhibits *APOC3* mRNA. In a phase 2b placebo controlled clinical trial involving 229 individuals with hypertriglyceridemia with TG level above 500 mg/dL it was demonstrated that subcutaneously injection of ARO-APOC3 (10, 25, 50 mg) led to reduction of Apoc3 level up to 77% and mean TG level to 57% decrease within 48 weeks. It is stated that adverse effects were not of serious concern and were not associated with treatment [106].

While CRISPR-mediated *APOC3* knockout in animal experiments demonstrates a significant reduction in TG levels and a decrease in atherosclerotic lesions, the application of this technology in humans faces several limitations, such as potential side effects and the need to develop safe delivery methods. In turn, siRNA-based therapy Plozasiran has already shown its effectiveness and safety in early clinical settings. However, the trial involved only two doses over 24 weeks, with monitoring extending to 48 weeks. More extensive and long-term investigations are required to evaluate the effectiveness and probable complications associated with treatment. Promising areas for further research include optimizing CRISPR/Cas9 delivery methods to minimize the immune response, as well as conducting large-scale, long-term clinical trials to evaluate the effects of APOC3 inhibition on cardiovascular events.

### 2.6. Microsomal Triacylglycerol Transfer Protein

Another potential gene therapy target for atherosclerosis is the gene encoding microsomal triacylglycerol transfer protein—Mtp. It encodes a protein which is necessary for the production of ApoB-containing lipoproteins, VLDL and chylomicrons assembly and intracellular TAG transport [107]. Deletion of the *MTP* gene in murine liver, as well as inhibition of the Mtp itself, leads to a decrease in plasma cholesterol and TAG levels, resulting in atherosclerosis regression [89]. It has also been shown that the expression of *Drosophila* Mtp in Mtp-deficient mice can mitigate liver steatosis [108]. Theoretically, reducing lipoprotein levels could be achieved by inactivating the microsomal triglyceride transfer protein gene (*MTP*) since this protein plays a crucial role in transferring triglycerides, cholesteryl esters, and phosphatidylcholine between membranes and is essential for the formation of apoB-containing lipoproteins [109].

It was shown that treatment with microRNA-30c (miR-30c), which interacts with the 3′-untranslated region of *MTP* mRNA, induces its degradation, leading to a decrease in Mtp activity and apolipoprotein B (ApoB) secretion. This, in turn, led to a decrease in total plasma cholesterol in the experimental group (~240 mg/dL vs. ~170 mg/dL in the control group) [110]. There was a study using Mtp inhibitor that can significantly reduce the presence of cholesterol in the blood in an atherosclerosis mouse model [111]. Mtp inhibitor BMS 212122 was studied on *LDLR*−/− murine model to investigate whether it promotes atherosclerosis regression from a short-term perspective [111]. The three groups of mice were involved in the experiment: baseline group, chow diet as a control and chow diet combined with inhibitor (25 mg/kg) during 14 weeks, eight animals in each group. Previously, the animals were on a Western diet for 16 weeks. The results demonstrated that the inhibitor reduced blood cholesterol level by 83% (201.1 ± 53.8 mg/dL) and by 94% (70.0 ± 11.8 mg/dL) compared to baseline, one and two weeks later, respectively. Mice placed on a chow diet with Mtp inhibitors reached cholesterol levels of wild-type mice (70 mg/dl), while the chow group cholesterol exceeded 250 mg/dL at the same period. There have been no major changes in aortic root plaque sizes. Lipid content in plaques was considerably reduced in the inhibitor group compared to baseline (26–29% of plaque area vs. 37.5–41.5%). However, nothing is stated about a decrease in plaque amount.

Aegerion Pharmaceuticals has developed a small-molecule drug, Lomitapide, which targets and inhibits MTP, thereby suppressing the synthesis of VLDL in the liver to a dose-dependent shortage of LDL cholesterol in patients with homozygous familial hypercholesterolemia.

It could be concluded that *MTP* could serve as a promising target for gene therapy of atherosclerosis and FH, aiming at the reduction of *MTP* product via gene editing, RNA interference or synthesis of antibodies binding to Mtp to prevent its activity.

### 2.7. Sterol Transporters

Sterol transporters are enzymes that contribute to the transport of cholesterol and thus should be considered as potential therapeutic targets for the reduction of cholesterol levels. microRNAs (miRNAs), small (usually with a length of 21–23 nucleotides) non-coding RNA that can be involved in RNA silencing and posttranscriptional regulation of gene activity, can also participate in lipid metabolism regulation. Namely, miR-33 inhibits the expression of sterol transporters genes *ABCA1* (ATP-binding cassette transporter A1) and *ABCG1* (ATP-binding cassette transporter G1) and reduces cholesterol efflux [112], which play an essential role in the atheroprotective reverse cholesterol transport [113]. Thus, the proper regulation of cholesterol efflux may help to reduce cholesterol blood levels, affecting atherosclerosis development. Therapy based on miRNA faces complex challenges such as off-target toxicity, unintended change of gene expression, severe immune responses, effects on non-target tissues and more. For these reasons, miRNA therapy is currently under development; only a few miRNA therapies have reached late-stage clinical trials.

### 2.8. Conclusion

Based on the evidence provided in Section 2, we consider the most promising ones to be *PCSK9*, *ANGPTL3*, and *APOC3*. siRNA-based drugs (ARO-ANG3) in phase I trials showed up to 80% reduction of blood Angptl3 and significant lipid lowering [97]. For comparison, Evinacumab, approved for homozygous FH, reduces LDL-C by 47%. Based on the results on *Macaca fascicularis*, which are closer to humans than mice (genetically and biochemically) there was 90% Pcsk9 and 60% cholesterol levels decrease [82], and in mice bearing human hepatocytes Pcsk9 was reduced by 52% on average from baseline [76]. VERVE-101 (LNP encapsulated base editor) reduced Pcsk9 by 84% and LDL-C by 55% in early clinical trials [85,86]. For *APOC3* there are trials on humans with promising results: Plozasiran phase IIb trials showed 77% Apoc3 reduction and 57% TAG reduction [106].

Lipid and lipoprotein metabolism, potential targets for gene therapy, are summarized in Table 2.

## 3. Alternative Gene Targets for Atherosclerosis Treatment

Hypertriglyceridemia leads to coronary heart disease and is characterized by elevated blood levels of VLDL TAG [114]. Therefore, it could be appreciating to manipulate with genes involved in regulation of blood TAG levels regulation, such as *TRIB1*, deficiency of which promotes atherosclerotic lesion formation in *LDLR*−/− mice [115] and is associated with hypertriglyceridemia and familial hypercholesterolemia, or PLTP (phospholipid transfer protein).

**Figure 3:**
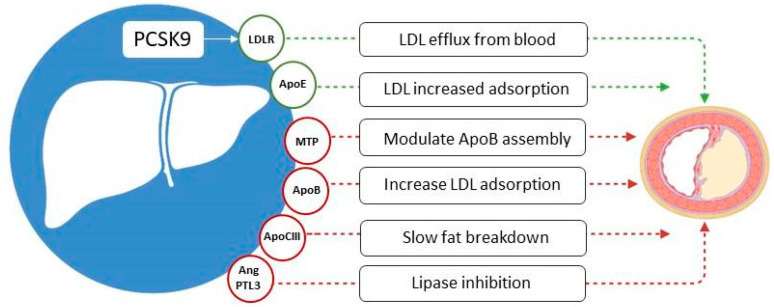
Potential gene therapy target genes related to lipid and lipoprotein metabolism. Red arrows originate from genes whose activity should be decreased in order to slow down atherosclerosis development. Green arrows originate from genes whose activity should be increased in order to slow down atherosclerosis development. The liver is the main organ where the expression of these genes should be modulated by gene therapeutics.

Mehrabian et al. demonstrated that *ALOX*−/− mice had an atheroprotective profile [116]. Previous studies provided the evidence that 5-lipoxygenase, which is involved in arachidonic acid processing and oxygenation, producing leukotrienes and lipoxins, is considered to contribute to atherosclerosis progression [117]. Since the whole picture of atherosclerosis pathogenesis is not clear yet, it is complicated to choose a convenient target for gene therapy. Recent studies have shown a link between ferroptosis and atherosclerosis. Alox5 is thought to be involved in the development of the necrotic core in atherosclerosis by participating in macrophage ferroptosis regulation [118]. Genes associated with ferroptosis and involved in atherosclerosis development, such as *HMOX1*, *NCF2*, *NOX4*, *MMP9*, *AKR1C3*, *ANGPTL7*, and *VLDLR*, were identified [118]. Although it is still required to make further investigations in this field, it directs us to potential targets for gene editing. We summarized some potential target genes related to lipid and lipoprotein metabolism, which could be used for gene therapeutics development (Figure 3).

### 3.1. Approaches of Gene Therapy Related to the Reduction of Inflammation Connected with Atherosclerosis

There is a well-established correlation between inflammatory processes and the formation of atherosclerosis. The inflammatory response plays a pivotal role in the development and progression of atherosclerosis.

Endothelial damage plays one of the main roles in atherosclerosis development [119]. Damaged endothelium may contribute to atherosclerotic plaque formation and development due to the inflammation process. Endothelial damage can be caused by a variety of factors such as smoking, high cholesterol and high blood pressure [120].

The damaged endothelium becomes more permeable to low-density lipoproteins (LDL), including different modified atherogenic LDL [121,122], which could enter the arterial wall and undergo oxidation [123]. Atherogenic LDL triggers an inflammatory response by attracting immune cells such as monocytes and macrophages to the site of injury [124]. These cells engulf the atherogenic LDL and turn into foam cells. Foam cells are a major component of atherosclerotic plaques. Inflammatory cytokines, such as IL1β and TNF-α, and other mediators released by immune cells contribute to further endothelial damage and progression of atherosclerosis. Thus, inflammation not only initiates the process of atherosclerosis but also supports its progression, making it an important target for the prevention and treatment of this disease.

Inflammation is a promising potential therapeutic target for the treatment of atherosclerosis. Firstly, inflammation promotes the development of atherosclerotic plaques, leading to narrowing of the vessel lumen [125]. Second, the process of inflammation attracts immune cells to the area of inflammation, leading to the release of cytokines and other inflammatory mediators, resulting in increased vascular damage. Thirdly, the development of inflammation, caused by cholesterol crystal accumulation, leads to an increased risk of atherosclerotic plaque dislodgement [126]. Cholesterol crystal accumulation causes inflammasome activation, which in turn activates IL-1*β* cleavage. It is the reduction of inflammation that leads to the stabilization of atherosclerotic plaques [127]. Fourthly, the development of inflammation leads to deterioration of the endothelial function of blood vessels [128]. Pro-inflammatory molecules, such as lipoproteins, interleukins and TNF-ɑ, can promote the expression of chemoattractants and adhesion molecules which help white blood cells to pass the endothelial monolayer [129]. Thus, a targeted treatment aimed at eliminating inflammation may improve the clinical picture in several ways.

One of the most cited and well-known studies on the role of inflammation in atherosclerosis is CANTOS (Canakinumab Anti-Inflammatory Thrombosis Outcome Study). A genetic polymorphism of the IL-6 signaling pathway has been shown to reduce plasma C-reactive protein levels [130]. One of the recent ways to fight atherosclerosis is the use of canakinumab antibody [131], assessed in clinical trials involving 10,061 patients. The main target of the canakinumab antibody is IL-1β. This cytokine is one of the major factors influencing the inflammation process. IL-1β regulates the expression of IL-6 and influences the increase in C-reactive protein levels. In this study, the researchers showed a significant decrease in C-reactive protein among the patients. In groups receiving Canakinumab, the median of C-reactive protein decline was 26–41% higher than in the control group. So, as we can predict, IL-1β is a potential target for anti-inflammatory treatment through knockdown.

The NLRP3 inflammasome plays an important pro-inflammatory role. The most important action of NLRP3 inflammasome in different diseases, including atherosclerosis, is activation of IL-1, through activation of inflammatory caspases and processing of proIL-β [132]. There are different ways of NLRP3 inflammasome activation [133]. Canonical ways, such as K^+^ efflux, Ca^2+^-involved activation, and lysosome leakage, are affecting NLRP3 inflammasome-dependent pathways. Non-canonical pathways of NLRP3 inflammasome activation also exist. Most investigated pathways are LPS-dependent. One of the non-canonical ways is the pathway of NLRP3 inflammasome activation through LPS-dependent caspase-11 activation. The alternative way is activated through binding LPS to TLR. It was already shown that different pathogens, including *Chlamidophila pneumoniae*, *Porphyromonas gingivalis*, *Helicobacter pylori*, influenza A virus, hepatitis C virus, cytomegalovirus, and human immunodeficiency virus, are increasing the risk of cardiovascular disease progression [134]. The NLRP3 pathway may be involved in atherosclerosis progression, and there is a lot of evidence that the IL-1β pathway is strongly involved in atherosclerosis progression. It was shown that NLRP3 inflammasome occurs in all the cell types during atherosclerotic plaque formation [135]. What is interesting is that there are different ways of inhibiting NLRP3 pathways. For example, a potential role of MCC950 as a NLRP3 inhibitor was shown [136]. Another potential way for NLRP3 inhibition is RNA interference by using lentiviral vectors [137] (in vivo experiments using murine models). Usage of such therapy showed atherosclerotic plaque stabilization. It was shown that silencing by miRNAs inhibited NLRP3-dependent pyroptosis [138] (in vitro experiments using cell lines). Another factor that can be a target for NLRP3 activation and IL-1β expression is *TFEB* (transcription factor EB) [139]. It was shown that overexpression of *TFEB* decreases the level of IL-1β by more than twofold. All this makes the NLRP3 inflammasome a potential target for silencing in cells involved in plaque formation.

Another interesting target to suppress the inflammatory response is the ABCA1 protein. The effect of *ABCA1* gene overexpression in endothelial cells was studied in vitro [140]. Overexpression of the *ABCA1* gene was shown to have multiple anti-inflammatory effects. When the *ABCA1* gene is overexpressed in endothelial cells, it decreases the levels of mRNA expression of interleukin-6, tumor necrosis factor, VCAM-1 protein, decreases the level of toll-like receptor 4, and leads to increased nitric oxide production. *ABCA1* gene overexpression has not been shown to increase cell migration or apoptosis. Overexpression of *ABCA1* in endothelial cells has also been shown to increase ApoAI-mediated cholesterol efflux. As it was already shown, an important role in ABCA1 activity plays NMDA receptors (N-methyl D-aspartate receptors (NMDAR)). NMDA receptors are ligand-dependent cationic receptors. Activation of NMDAR initiates several signaling pathways, where one of the most important is cation influx. NMDARs play an essential role in synaptic transport [141]. But NMDARs are highly expressed not only in neural cells. It was shown that NMDARs are expressed already in central and peripheral glial cells, endothelium, kidney, pancreas, bone [142] and other types of cells, including macrophages too [143]. As it was shown, they play a crucial role in ABCA1 proteolysis) [144]. It was shown that overexpression doesn’t affect *ABCA1* mRNA expression level, but it does affect the final protein level because of increased activity of calpain, a highly productive protease. Thus, we can conclude that overexpression of NMDARs affects cholesterol efflux and the formation of foam cells. Cholesterol efflux by cells, in turn, leads to a decrease in lipid accumulation in vascular cells, which in turn is one of the causes of atherosclerosis. All this makes *ABCA1* a potential target for atherosclerosis treatment. It may be of interest to increase the level of *ABCA1* expression with the use of AAV vectors.

One more potential target for anti-inflammatory treatment is the *NDRG1* gene. The *NDRG1* gene is mostly linked with oncogenic processes. One of the important roles of *NDRG1* is preventing tumor growth and suppressing metastases [145]. The Ndrg1 protein plays an important role in cell differentiation and growth. The Ndrg1 protein is also activated under various stress conditions [146]. Previously, the *NDRG1* gene was shown to play an important role in the development of cancer [147]. A more recent study has shown that the Ndrg1 protein also plays an important role in inflammatory processes. *NDRG1* expression was shown to be significantly upregulated by ≈3.5-fold in atherosclerotic lesions of human and mouse endothelial cytokine-stimulated cells during treatment with IL-1β [148] (in vivo experiments on murine models and in vitro on human cell lines). Knockdown of the *NDRG1* gene using a lentiviral vector containing hairpin RNA significantly reduced (inhibited 80% reduction of protein expression, as shown by Western Blot analysis) the expression of Ndrg1 protein during IL-1β and TNF-α treatment [148]. Additionally, it was shown that knockdown of *NDRG1* suppresses cellular adhesion by 70%. In addition, knockdown of the *NDRG1* gene was shown to have an antithrombotic effect by decreasing the expression of procoagulant molecules and increasing the expression of thrombomodulin and vWF (von Willebrand factor [149]. And as we can see, *NDRG1* is a really important gene, involved in the inflammatory process, which makes it a potential target for gene therapy. For example, knockdown of *NDRG1* in EC involved in atherosclerotic plaque formation could decrease the pace of plaque growth through new cell adhesion.

Another potential target for silencing is lncRNA (long non-coding RNA) *TUG1* (taurine upregulated gene 1). It is interesting that lncRNA *TUG1* has different possible pathways of atherosclerosis progression. It was shown, that *TUG1* knockdown decreases level of total cholesterol (TC) (decreased by about 30% compared to the control group), triglyceride (TG) (decreased by about 50% compared to the control group), low-density lipoprotein cholesterol (LDL-C) (decreased by about 30% compared to the control group) and high-density lipoprotein cholesterol (HDLC) (decreased by about 50% compared to the control group) in serum [150](in vivo experiments using murine model). At the same time, it was shown that *TUG1* knockdown causes a decrease in IL-6 (decreased by about 30% compared to the control group) and TNF-α (decreased by about 50% compared to the control group). It was shown that expression of lncRNA *TUG1* causes boosted proliferation, elevates inflammatory factors expression and suppresses apoptosis through silencing miR-133a. What is important, miR-133a plays a crucial role in *IGF-1* (insulin-like growth factor-1) expression and proliferation of vascular smooth muscle cells [151]. Another pathological pathway of lncRNA *TUG1* is atherosclerosis promotion through affecting miR-92a. MiR-92a is a factor that is predicted to affect the *ApoM* expression level [152]. It was shown that *TUG1* silenced miR-92a, which enhances *FXR1* expression and downregulation of *ApoM*. ApoM is an important factor in atherosclerosis pathogenesis. Predicted role of ApoM is producing larger particles of HDLC, which exhibits an atherogenic effect [153]. All aforementioned facts suggest that *TUG1*′s role in atherosclerosis pathogenesis makes it a potential therapeutic target through knockdown.

Macrophages play a key role in activating or resolving inflammatory processes, producing different factors, which are important for wound healing and in clearing the wound area from cell debris [154]. Modern researchers prefer to categorize macrophages into two main groups: M1 and M2 macrophages [155]. As discussed in different articles, M1 and M2 macrophages play divergent roles in human organisms. M1 macrophages are mostly called proinflammatory cells, while M2 macrophages undertake healing and anti-inflammatory roles. Both of them, as it is commonly believed, proliferate out of M0 macrophages, which are primarily silenced in the body. Different factors can cause the process of differentiation into M1 and M2 macrophages. For example, a high level of LPS (lipopolysaccharide, mostly out of microbial cells) and IFN-γ drives the differentiation to M1 phenotype, while the increased level of IL-4 (interleukin 4) causes the M2 macrophages polarization [156]. Actually, there is an important phenotypic difference between M1 and M2 macrophages. When polarized, M1 macrophages secrete IL-1β, IL-8, IP-10, IFN-γ, TNF-α, and CCL5, while M2 macrophages secrete IL-13, CCL17, and CCL18 [157]. One of the more interesting perspectives is the way of switching macrophages from one phenotype to another. As it was found in experiments using ex vivo isolated cell lines, five transcriptional factors (CTCF, E2F1, MYC, PPARγ, and STAT6) form a molecular signature of M2 macrophages [158]. Knocking down the expression of these factors switched the M2 macrophages to pro-inflammatory M1 macrophages. And, actually, the reverse process is possible. It was shown that adding to M1 macrophage culture M2-promoting stimuli such as IL-4 causes the switch from M1 to M2 phenotype [159]. It was shown that the level of CD206 expression was increased and the level of CCR7 reduced. It was shown that expression of M2 markers such as CCL18, MDC/CCL22, CD206/MRC1, PDGF, and TIMP3 increased. Exosomal-guided switch is a potential way for macrophages to reprogram [160]. So, as we can see, changes in macrophage phenotype play an important role in the inflammatory process, and it can be a potential therapy target, because the decrease of inflammation via the macrophages is one of the potential ways to fight atherosclerosis disease. As a potential target, we can suggest reprogramming M1-macrophages into the M2 phenotype. This can be reached in two ways. First of all, we can knock down factors involved in the formation of the M1-phenotype with the use of lentiviral constructs, containing silencing RNA. The second way is to overexpress the transcriptional factors, which are involved in M2-phenotype formation. This can be reached by the use of AAV vectors.

What else is interesting is that macrophages in atherogenic plaque undergo RIPK3 (receptor-interacting serine/threonine-protein kinase 3)-MLKL (mixed lineage kinase domain-like protein)-dependent necroptosis [161]. The leading role in this process is played by *RIPK1* (receptor-interacting serine/threonine-protein kinase 1). This enzyme plays an important role in controlling whether a cell undergoes inflammation-dependent, caspase-dependent apoptosis or necroptosis under the influence of extracellular factors. It was shown that the *RIPK1* gene expression shows a high correlation with early stages of atherosclerosis in humans. *RIPK1* gene expression has been shown to be differentially expressed in M1 and M2 macrophages in atherosclerotic lesions in both humans and mice [162]. It was shown that whereas Ripk1 levels in M2 macrophages were unchanged, a 3.2-fold increase in *RIPK1* gene expression in M1 macrophages relative to non-treated M0 macrophages was shown. It was also shown that knockdown of *RIPK1* in endothelial cells prevented translocation of NF-κB to the nucleus in response to TNFα, resulting in decreased gene expression of *IL1B*, E-selectin, and causing apoptosis progression [163]. All the above raise the interest around *RIPK1* as one of the most important genes in the inflammatory process.

Macrophages play one more important role in the initial stages of atherosclerosis. Uptake of LDL in macrophages and smooth muscle cells (SMC) causes the development of foam cells. Actually, the development of foam cells is the first symptom of atherosclerosis [164]. By use of computational analysis, it was shown that there is a correlation between SMC and macrophages. 15 genes (*CTSD*, *HHEX*, *TNFRSF21*, *GLRX*, *EDEM2*, *CTSC*, *LAT2*, *SPOCD1*, *ABCA1*, *LST1*, *CD74*, *PLAUR*, *BRI3*, *EMB*, and *RNF13*) where identified as common for SMC and macrophages. But only three genes (*ABCA1*, *GLRX* and *RNF13*) are involved in processes of foam cell formation [165]. The correlation in common gene expression in plaque development involving SMC and macrophages provides interest in further research of foam cell formation and the ways of avoiding their development. Actually, one of the potential ways of study is understanding which types of macrophages are involved in foam cell development and which potential ways for preventing foam cell development we can find. For example, as was already told, *ABCA1*, being involved in cholesterol efflux, is a suggested potential target for overexpression in foam cells, to reduce cholesterol accumulation inside these cells and avoid their growth. An overexpression of *ABCA1* can be reached with the use of AAV vectors. Potential gene targets to delay/prevent atherosclerosis via the inflammatory pathway are summarized in Table 3. It should be mentioned that so far, one of the most promising targets of the inflammatory pathway in atherosclerosis is IL-1β, since this target has been explored in clinical trials. However, considering the involvement of IL-1β in many inflammation-related processes, the development of a precise gene therapeutic approach seems to be quite a challenging task.

### 3.2. Gene Therapy of Atherosclerosis Targeting Non-Coding RNA

In recent years, non-coding RNAs (ncRNAs) have attracted considerable research interest because of their impact on cardiovascular disease, providing potential avenues for developing personalized medicine and multiple therapeutic approaches. MicroRNA, lncRNA and circRNA are involved in the development of atherosclerosis (AS), regulating proliferation, cell activation, adhesion and the production of inflammatory factors in endothelial and vascular smooth muscle cells.

Natural antisense transcripts (NATs) represent one of the major RNA groups in mammalian genomes and are considered to be a subclass of long non-coding RNAs [166]. NATs are transcribed from the reverse strand of protein-coding genes. This approach is of interest for diseases that require regulation of “untreatable” targets: transcription factors or growth factors [167]. Targeting blocking NATs increases their expression, benefiting potential therapy approaches. Compared to transcription factors, NATs also exert their effects more quickly and act faster due to their close proximity, allowing them to adapt immediately [168].

lncRNA nucleoside diphosphate-linked moiety X motif 6 (*NUDT6*) has been found to act as a natural antisense transcript (NAT) to fibroblast growth factor 2 (*FGF2*) [169].

The Fibroblast Growth Factor 2 (FGF-2 or bFGF) belongs to the heparin-binding protein family and is involved in the pathogenesis of atherosclerosis and also has cardioprotective effects [170]. FGF-2 has an effect on the differentiation, proliferation and migration of vascular cells. Its expression in the normal vascular wall is useful for maintaining vascular homeostasis and protecting endothelial cells [171]. In all this, FGF-2 and its receptors take part in the inflammatory process by accelerating the growth of atherosclerotic plaques while stimulating the proliferation and migration of vascular smooth muscle cells [172,173].

The role of *FGF2* in atherosclerosis is dynamic and depends on the stage of the disease. It was shown in *ApoE–/– FGF2*–/– mice models that knockout of low molecular weight *FGF2* inhibits atherosclerotic progression at various stages [174]. In combination with PDGF-BB, FGF2 can normalize the structure of abnormal new blood vessels and thus improve the stability of the plaque [175].

Locked nucleic acids (LNA) of site-specific ASO (GapmeRs) were delivered to carotid plaques in an atherosclerotic ApoE-deficient mouse model. The engineered GapmeR targeted *NUDT6* in exon 2, which does not overlap with Fgf2. Inhibition of Nudt6 in the inducible plaque rupture in vivo model led to a reduction in the rupture rate from 60% to 25%.

Treatment with *NUDT6* ASO (GapmeR) resulted in a slower increase in abdominal aortic diameter (1.6 mm in the negative control group and 1.3 mm in the *NUDT6*-ASO group) over a treatment period of up to 28 days) [169].

Thus, site-specific ASO (GapmeR) silencing of *NUDT6* expression reduced plaque rupture rates and experimental abdominal aortic aneurysm growth in preclinical disease models, which are closely related to atherosclerosis burden and vascular inflammation in the infrarenal portion of the aorta. These data provide an opportunity to develop new therapeutic strategies to limit the burden of vascular disease [169].

The next step of this study was to work with *LDLR*−/− Yucatan minipigs as an in vivo preclinical model of abdominal aortic aneurysm to demonstrate the feasibility of enhanced *NUDT6* inhibition. This is the first study in the field of cardiovascular disease to target an lncRNA with an ASO in a large porcine animal model.

To avoid unwanted off-target effects of ncRNA modulation, it is necessary to develop effective targeted delivery methods. Researchers often use nanoparticles as scavengers to deliver therapeutic agents, particularly nucleic acids.

It is now known that multiple AAV serotypes can be used for tissue-specific delivery based on natural tropism for certain cell types and interactions between different cellular receptors and serotypes. Tissue- or cell-specific delivery can also be enhanced by providing tissue-specific promoters for tissue-specific expression of miRNA. AAV-based constructs are already being used in gene therapy and many other clinical trials, with encouraging safety profiles.

Some ncRNAs have been implicated in AS and are being used as markers for early diagnosis and potential therapeutic targets [14].

It was found that LncRNA myocardial infarction-associated transcript (*MIAT*) was significantly elevated in advanced AS mouse models [176]. 2.5-fold increased expression of LncRNA *PVT1* was observed in patients’ serum with coronary AS, which was a risk factor for CAD [177]. More than 8 times the magnification of miR-106b in the plasma of 45 patients has been determined to be closely associated with AS [178]. But, only a limited number of studies have identified a role for PVT1 in the pathogenesis of AS through the regulation of miR-106b.

Ferroptosis is a new type of programmed oxidative necrotic cell death based on iron-dependent lipid peroxidation [179]. Judging by recent studies using mice and rat models, ferroptosis is associated with the progression of AS [180,181].

One of AS’s main problems is fatty build-up in artery walls, with proliferation of smooth muscle cells and fibrous matrix, leading to progressive plaque formation. Ferroptosis is caused by intracellular free iron interacting with hydrogen peroxide via a Fenton reaction, resulting in plasma membrane polyunsaturated fatty acid degradation [182]. In addition to reducing lipid peroxidation and iron content in murine aortic endothelial cells, these compounds also ameliorate cellular lipid peroxidation and endothelial dysfunction, and attenuate AC injury [183].

It has already been proven that *PVT1* can regulate ferroptosis [184]. But until a certain point, it remained unknown whether *PVT1* could regulate ferroptosis in HUVEC, contributing to the onset and development of AS.

LncRNA *PVT1* had affected ox-LDL-induced HUVECs, leading to a significant 4-fold increase in the expression of ferroptosis markers *ACSL4* and *PTGS2*, and a reduction by half in miR-106b-5p signaling and contribution to atherosclerotic plaque formation [185].

The experiment also included in vivo experiments with *ApoE*−/− mice on a Western diet, and it was noted that the number and size of atherosclerotic plaques in the aortic arch increased. Next, PVT1, PBS, si-NC, or si-LncRNA PVT1 were administered to *ApoE*−/− mice for 12 weeks. As a result, the expression of LncRNA *PVT1* in the si-LncRNA PVT1 group was significantly reduced by almost 70%.

Aortic arch of *ApoE*−/− mice transduced with si-LncRNA PVT1 showed decreased number and size of atherosclerotic plaque compared with the si-NC group, by 70% [185].

The suppression of LncRNA *PVT1* significantly promotes the progression of AS mediated by miR-106b-5p/ACSL4 [185]. Thus, LncRNA *PVT1* could be a potential therapeutic target for AS. Currently, spinal muscular atrophy and Duchenne muscular dystrophy are treated with antisense technology. Transfection and invasive tail injection of siRNA into mice were used in this report. We suggest using AAV as a delivery system to improve specificity, as in the example above.

MiRs are small, specifically expressed non-coding RNAs that act as transcriptional and post-transcriptional regulators of gene expression and play important roles in regulating cellular processes [186]. New studies show miRs to be novel biomarkers of cardiovascular diseases, including atherosclerosis, diabetes, acute myocardial infarction and stroke [187,188,189].

Moreover, it has become increasingly clear that microRNA-mediated gene expression in endothelial cells represents a crucial mechanism underlying cardiovascular homeostasis. An increasing number of studies indicate that microRNA-1 and microRNA-210 play a role in regulating endothelial cell function, cytokine responses and vascular inflammation in atherosclerotic disease [190,191,192]. Inhibition of miR-17 has been shown to have a protective effect against atherosclerosis through reduction of inflammation and lipid accumulation in atherosclerotic lesions [193]. The role of miR-19a-3p has been unclear because, according to different researchers, miRNA expression and regulation may increase, decrease or remain unchanged in cardiovascular disease and vascular endothelial cells [194,195,196,197].

In vivo experiments had led to the identification of junctional coronary artery disease-associated proteins (JCAD) as a novel target of miR-19a-3p, finding that miR-19a-3p levels were lower in tissues from mice with atherosclerotic coronary artery disease than in tissues from control mice [198]. The miRNA expression profile in early-stage murine atherosclerotic plaques compared to intact arterial tissue was analyzed and presented in the form of heat maps to investigate the role of miR signatures in the progression of atherosclerosis. After 3 months of a fatty diet, the expression of miR-19a was lower in atherosclerosis than in healthy arterial tissue. Furthermore, MiR-19a expression was significantly lower in arteries of mice fed a fat-rich diet for 10 months than in arteries of mice fed a fat-rich diet for 3 months [198].

Based on these findings, the researchers speculated that miR-19a could be a potential therapeutic target for preventing the progression of atherosclerosis [198]. To further investigate its role, HUVEC cells were transfected with Ad-JCAD, miR-19a-3p mimic and miR-19a-3p mimic + Ad-JCAD. The transfection of Ad-JCAD led to a 5-fold increase in the expression level of JCAD, while the inhibitory effect of miR-19a-3p was reversed to the control level.

In summary, the miR-19a-3p/JCAD axis is involved in the modulation of inflammation and endothelial cell proliferation. This axis represents an important regulatory mechanism in endothelial function and the pathogenesis of atherosclerosis, providing new knowledge about potential therapeutic strategies.

All the options discussed above are potential targets for the treatment of atherosclerosis due to their involvement in the pathogenesis of the disease. We proposed to use AAV as a delivery vehicle, due to the small size of non-coding RNAs, we can avoid one of the main limitations of AAV—capsid capacity. Several researchers also came to this conclusion and tested the AAV delivery technique.

The long non-coding RNA (lncRNA) LeXis (liver-expressed LXR-induced sequence) promoted interaction between the liver X receptor and the SREBP transcription factors to maintain liver sterol content and serum cholesterol levels. AAV8 vector was used to express the LeXis under the control of the thyroxine-binding globulin (TBG) promoter from the human liver [199].

To clarify whether LeXis gene therapy possesses an effect on AS, en face lesion analysis was carried out. *LDLR*−/− animal treatment with AAV8.hTBG.LeXis demonstrated a reduction of almost 2 times in AS load compared to mice in the control group. Moreover, serum lipid analysis revealed a significant decrease in total cholesterol and triglyceride levels in mice treated with LeXis [199].

AAV8.hTBG LeXis administration resulted in sustained expression of LeXis and a substantial decrease of 25% in *SREBP2* and its target genes (*SQLE* by 50%) involved in cholesterol biosynthesis, including *HMGCR* in mouse liver [199].

The obtained data indicate the feasibility of a lncRNA mimetic therapeutic strategy for atherosclerosis. Some new treatment agents have shown significant efficacy in lowering cholesterol levels, but have raised concerns about the long-term development of fatty liver and hepatotoxicity. Although the degree of atherosclerosis reduction was modest [199], evidences of hepatotoxicity were not observed. Moreover, the efficacy was similar to miRNA-based therapy for atherosclerosis.

An example of miRNAs participating in lipid metabolism is miR155, but its role in atherosclerosis progression is ambiguous. In mice carrying *ApoE* deletion (*ApoE*−/−), inhibition of miR155 resulted in atherosclerotic plaque regression, whereas *LDLR*−/− mice lacking miR155 demonstrated the opposite effect [200]. For the purpose of RNAi-mediated gene silencing, it may be suitable to apply a retroviral vector system for the delivery of siRNA and miRNA. There are some challenges for siRNA delivery, such as degradation by nucleases. RNA nanotechnology is a novel approach to achieving successful therapy and addressing obstacles. For instance, it may be possible to use polymers as a platform for siRNA delivery, for instance, polyethylenimines [201]. Extracellular vesicles are also widely used as platforms for small RNA delivery for therapy. Moreover, there have been designed tissue/organ specific EVs [173]. Exosomes carry miRNA, protect it from nucleases and promote RNA stability [202].

**Figure 4:**
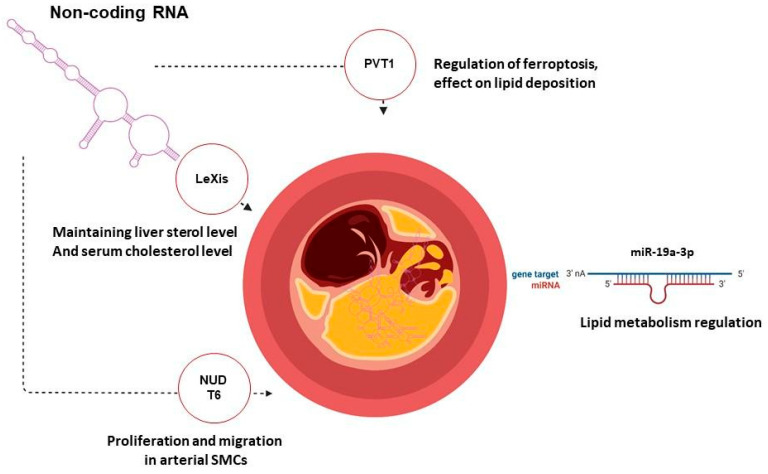
Types of non-coding RNAs with a potential for gene therapy and their role in the pathogenesis of atherosclerosis.

Figure 4 summarizes a few non-coding RNAs and briefly depicts their role in the pathogenesis of atherosclerosis. Some non-coding RNAs could potentially be used as gene therapeutics if their mechanism of action is thoroughly elucidated.

Table 4 summarizes non-coding RNA that could have been used in the gene therapy of atherosclerosis.

## 4. Conclusions and Future Perspectives

As we can see, multiple genes have already been identified as potential targets for gene therapy of atherosclerosis, with different stages of research and clinical investigations. However, the complexity of this disease makes it hard to create an ideal drug capable of helping everyone to prevent or delay atherosclerosis development. Many different pathological aspects that seem to contribute to atherosclerosis development make it hard to find the main one (or combination of them), which is at the root of the development of this disease. This makes finding a cure for atherosclerosis (including gene therapy) a challenging task.

We should address why mitochondrial genes cannot yet be included as targets for gene therapy in atherosclerosis. It should be noted that a correlation between mitochondrial mutations and the occurrence of atherosclerosis has been observed [203]. Also, in 2016, a study on a line of C57BL/6J mice with *ApoE*−/− gene deficiency revealed that atherosclerosis is a consequence of mitochondrial mutations [204]. Mutations of mitochondrial DNA negatively affect the processes of oxidative phosphorylation and transcription in mitochondria. It subsequently leads to increased oxidative stress, as well as to the occurrence of inflammatory processes and metabolic disorders [205]. Mitochondrial mutations in several genes, such as *rRNA12S*, *UUR* and *CUN* recognizing codon of the tRNA-Leu, subunits of the respiratory chain cytochrome B and 1, 2, 5, 6-NADH dehydrogenase, are associated with atherosclerosis and the formation of atherosclerotic plaques [206]. However, additional research (especially in order to identify the location of mitochondrial mutations on the axis “reason-consequence” for atherosclerosis) is needed before choosing the most promising mitochondrial targets for gene therapy of atherosclerosis. Also, the correction of the mitochondrial genome so far is an even more challenging task than the correction of the nuclear genome, and without robust instruments for mitochondrial DNA editing, it is hard to proceed further in this direction.

Due to the high complexity and potential risks associated with gene therapy, specialized regulatory pathways are required [207]. A key prerequisite for successful gene therapy is screening patients’ blood for the presence of antibodies that could interact with the therapeutic vector, for example, detecting anti-AAV capsid antibodies prior to treatment. Additionally, modulation of the adaptive immune response, such as through the use of mTOR inhibitors, may be necessary to enhance therapeutic efficacy [208].

Despite the challenges inherent to gene therapy, including those related to gene editing approaches discussed earlier in this review, there are notable advances in the field. For instance, CRISPR-Cas9 gene therapy has achieved regulatory approval, as exemplified by Casgevy for the treatment of sickle cell disease [209].

One of the important aspects of gene therapy is the ethical considerations. Major ethical and social issues include religious and philosophical objections, lack of consent from future generations, stigmatization of people with disabilities, social inequality, parental pressure, unpredictable consequences, and the potential for misuse.

Somatic gene editing, which targets individuals already born, is generally considered easier to regulate and raises fewer ethical concerns than germline (heritable) editing. Germline editing introduces changes that can be passed to future generations, raising significant questions about consent, long-term monitoring, and intergenerational impacts. International approaches vary: the European Convention on Human Rights and Biomedicine prohibits heritable genome editing, while in the United States, such editing is not explicitly banned but is not considered by the FDA. In China, regulatory attitudes have been more permissive, but high-profile cases have highlighted ongoing ethical concerns and the need for robust oversight. Overall, ethical governance, social justice, and broad societal dialogue are recognized as essential for the responsible advancement of gene therapy [210,211].

Gene therapy in pediatric populations presents complex ethical challenges [212]. Many genetic diseases manifest early in life, significantly impacting children’s quality of life and life expectancy, making gene therapy a potentially transformative intervention. However, the risks—including immunogenicity, toxicity, and unpredictable long-term effects—must be carefully evaluated, as children may respond differently to therapies that have been clinically tested in adults due to physiological differences.

Additionally, the ethical oversight of pediatric gene therapy is critical. Ethics committees must ensure that protocols protect the best interests of the child, balance potential benefits and harms, and provide equitable access to research opportunities. High-profile adverse events, such as deaths following gene therapy administration—for example, acute liver failure reported after Elevidys (delandistrogene moxeparvovec) treatment for Duchenne muscular dystrophy—underscore the need for rigorous safety monitoring and transparent communication with families about potential risks.

In summary, gene therapy in children involves unique ethical considerations regarding informed consent, risk assessment, physiological differences, and the need for robust ethical oversight to safeguard vulnerable pediatric populations.

Another important aspect of gene therapy is its high cost [213]. One of the most widely recognized and expensive gene therapies is onasemnogene abeparvovec, used for the treatment of spinal muscular atrophy, with a single dose costing approximately €1,945,000 [214]. The primary drivers of these high costs include the complexity of production technologies, the limited patient populations associated with rare diseases, which hinder economies of scale, the single-use nature of most gene therapies, a lack of market competition, and the proprietary nature of manufacturing processes. Despite these substantial costs, gene therapy offers the potential for transformative, and in some cases curative, treatment for many serious conditions. In the long term, such therapies may also reduce the overall healthcare burden by eliminating or significantly reducing the need for ongoing, conventional treatments.

At the moment, many studies have been conducted that indicate the effectiveness of gene therapy in the context of treating atherosclerosis, and the factors involved in the pathogenesis of this pathology have been identified. However, there are a number of difficulties that hinder the development of gene therapy and its implementation in clinical practice. The main difficulty is identifying the root cause of the disease, which will eliminate it at the root, and not delay its inevitable appearance, eliminating the symptoms. Atherosclerosis is a complex pathology, the development of which is facilitated by both lipid metabolism disorders and inflammatory processes, but at the moment, there is no consensus on identifying the “starting point” in these processes. Experiments with changing the expression of apolipoproteins show encouraging results, but the lack of understanding of their interaction with each other and with other metabolites complicates the choice of a specific target for the effective action of gene therapy. For example, reducing ApoB expression using RNAi-based technologies reduces plasma cholesterol levels, but knocking down ApoB mRNA also reduces the expression of ApoB-48, a natural alternative isoform of ApoB required for chylomicron assembly in the intestine, which may lead to possible downstream unwanted side effects such as fat malabsorption or steatorrhea. This calls for new methods to improve the precision of gene editing to target specific exons. It should also be noted that while studies typically focus on the effect of a specific apolipoprotein on cholesterol or LDL-C, it would be equally important to conduct studies that simultaneously alter the gene expression of multiple apolipoproteins to better understand their interactions in the context of atherosclerosis. Also, further research limits the current capabilities of vectors for delivering target genes. Designs based on adeno-associated viruses have long proven themselves due to their low immunogenicity, as well as wide tropism capabilities for various tissues, but at the same time, they have a fairly low capacity. When choosing a vector, it is necessary to take into account the limited packaging capacity of the AAV vector (~4.7 kb). Several solutions have been developed to overcome the limitations associated with the delivery of bulk genes via AAV, such as: trans-splicing AAV vectors, proteasome inhibitors, fragmentation of the transgene, capsid engineering, and usage of multiple AAV vectors [215]. There are also liver-specific promoters that successfully work within the limited capacity of AAV, for instance, ApoE-hAAT. One of the alternatives is the use of lentiviral vectors. They have a larger capacity (~8–9 kb) and demonstrate stable liver gene transfer [216]. A new trend in the development of viral vectors could be the development of chimeric hybrid vectors that combine the properties of various systems. Another promising direction can be considered the use of various microRNAs and antisense RNAs. Due to their small size, it is possible to create complex expression cassettes within the existing AAV vectors, allowing for the influence of the expression of several lipid metabolism metabolites at once, for example, to create a knockdown of a number of apolipoproteins, which was mentioned above. In the field of non-viral vectors, recent advances in nanotechnology have led to the creation of promising and more effective delivery systems, but there are still problems with the stability of these particles and the possibility of delivery to the cell nucleus.

An equally important problem in the development of atherosclerosis treatment, as well as gene therapy in general, is that gene delivery approaches in animal models do not always predict a similar outcome for humans. This creates a demand for the creation of reliable animal models for preclinical evaluation of future gene therapeutics.

## Figures and Tables

**Table 1:** Types and functions of LDL in blood plasma.

Type	Molecule Size, nm	Function and Role in the Development of Atherosclerosis
Low-density lipoproteins (LDL)	18–26	Transport of cholesterol, triacylglycerides and phospholipids from the liver to peripheral tissues. After penetrating the subendothelial space, it can be subjected to oxidative modification. Oxidized LDL and LDL with other modifications can be captured by: 1. smooth muscle cells of the vascular wall via scavenger receptors, 2. macrophages, which are transformed into foam cells overloaded with cholesterol esters.
Intermediate-density lipoproteins (IDL)	25–35	Transport of cholesterol, triacylglycerides and phospholipids from the liver to peripheral tissues. Being intermediate particles in LDL metabolism, also contribute to the accumulation of cholesterol in the vascular wall. They participate in the formation of the lipid core of the future atherosclerotic plaque, enhancing the processes of cellular infiltration and inflammation.
Very low-density lipoproteins (VLDL)	30–80	Contains apolipoprotein B 100 (Apo B-100), which transports cholesterol, triacylglycerides and phospholipids from the liver to peripheral tissues. During lipolysis, under the action of lipoprotein lipase, VLDL is transformed into residual particles that are easily retained in the subendothelium and undergo modifications. The accumulation of foam cells, cellular decay products and extracellular lipid material leads to the formation of a necrotic core of an atherosclerotic plaque—a key element of unstable lesions.

**Table 2:** Potential targets related to lipid and lipoprotein metabolism for gene therapy of atherosclerosis.

Target Gene	Approach	Model/Study	Key Findings	Reference
*LDLR*	Exosome-delivered *LDLR* mRNA	*LDLR*−/− mice (FH model)	- Reduced serum cholesterol by 50% and atherosclerotic plaques by 3-fold.	[26]
*LDLR*	CRISPR/Cas9 editing via AAV8	*LDLR* E208X mice	- Partial Ldlr restoration (11% of WT); 2-fold reduction in plaque area.	[46]
*ApoB*	shRNA-mediated silencing (AAV8)	C57BL/6 mice	- 95% reduction in *ApoB* mRNA; 79% lower cholesterol.	[48]
*ApoE*	Lentiviral ApoE mimetic peptide	*ApoE*−/− mice	- 24% reduction in aortic lesions without cholesterol changes.	[56]
Lipoprotein (a) (Lp(a))	Antisense oligonucleotides	Phase 2b clinical trials completed	Reduction of Lp(a) by 35–58% in clinical trials	[53]
*PCSK9*	CRISPR/Cas9 (AAV8-SaCas9)	C57BL/6 mice	- 80% reduction in Pcsk9 protein; 35% lower cholesterol.	[71]
*PCSK9*	Lipid nanoparticles (base editing)	Non-human primates	- 90% *PCSK9* knockdown; 60% LDL-C reduction.	[82]
*PCSK9*	Inclisiran (siRNA)	Approved for clinical use in the EU (2020), UK and US (2021), China (2023). ORION-4 clinical trials are expected to be finished by 2049	- 51% LDL-C reduction in ASCVD patients with biannual dosing.	[68]
*ANGPTL3*	CRISPR/Cas9 (AAV9)	C57BL/6 mice	- 65% reduction in Angptl3; 57% lower LDL-C.	[78]
*ANGPTL3*	siRNA (ARO-ANG3)	Clinical trials (phase 1 basket trial)	- 80% Angptl3 reduction; significant LDL-C and TAG lowering.	[97]
*APOC3*	CRISPR knockout	Syrian hamsters	- Reduced atherosclerotic lesions and plasma TAG.	[102]
*APOC3*	siRNA (ARO-APOC3)	Clinical trials (2b placebo-controlled)	- 77% Apoc3 reduction; 57% TAG decrease.	[106]
*APOC3*	Volanesorsen (antisense oligonucleotide)	Approved for clinical use in the EU (2019)	Blood TAG levels decreased by 77%	[105]
*MTP*	miRNA-30c (inhibits MTP/APOB)	*ApoE*−/− mice	- Reduced plasma cholesterol (~30%) and ApoB secretion.	[110]
*MTP*	Small-molecule inhibitor (BMS 212122)	*LDLR*−/− mice	- 94% cholesterol reduction; reduced plaque lipid content.	[111]

**Table 3:** Potential targets of gene therapy related to the reduction of inflammation to affect atherosclerosis.

Target	Method	Model	Results	Reference
IL-1β	Antibody therapy	Clinical trials (10,061 patients)	Affecting IL-1β-regulated *IL-6* expression and decreasing the level of C-reactive protein expression. In groups receiving Canakinumab, the median of C-reactive protein decline was 26–41% higher than in the control group.	[131]
*NLRP3*	RNA-interference using lentiviral vector delivery	Murine model	Silencing of the *NLRP3* gene halted plaque progression and suppressed the expression of pro-inflammatory cytokines. RNA interference decreased macrophage and lipid content within the plaques, while increasing the presence of smooth muscle cells and collagen, thereby contributing to the stabilization of atherosclerotic plaques.	[137]
*NLRP3*	Increasing miRNA expression level using synthesized oligonucleotides	Cell lines	It was shown that the addition of miR-30c-5p reversed LDL-induced pyroptosis in the HAEC cell line. The abundance of *NLRP3* was decreased at the protein and mRNA levels.	[138]
*NDRG1*	RNA-interference using lentiviral vector delivery	Murine models and human cell lines	Knockdown of *NDRG1* using a lentivirus encoding NDRG1 shRNA reduces IL-1β- and TNF-α-induced expression of cytokines, chemokines, and adhesion molecules. NDRG1 inhibition also significantly decreases the expression of procoagulant factors such as plasminogen activator inhibitor-1 (PAI-1) and tissue factor (TF), while enhancing the expression of antithrombotic molecules like thrombomodulin (TM) and tissue-type plasminogen activator (t-PA), thereby promoting strong antithrombotic effects in endothelial cells.	[148]
lncRNA *TUG1*	RNA-interference using siRNA against *TUG1*	Murine models	Silencing of *TUG1* reduced hyperlipidemia, suppressed inflammatory responses, and alleviated atherosclerotic lesions in HFD-treated *ApoE*−/− mice. Over-expression of *TUG1* promoted cell proliferation, enhanced inflammatory cytokine production, and inhibited apoptosis in ox-LDL-exposed cells	[150]

**Table 4:** Non-coding RNA, which could have been used in gene therapy for atherosclerosis.

Target Gene	Approach	Model/Study	Key Findings	Reference
Nucleoside diphosphate-linked moiety X motif 6 (*NUDT6*)	Antisense oligonucleotides	*A*therosclerotic ApoE-deficient mouse model	Site-specific antisense silencing of *NUDT6* expression reduced plaque rupture rates and experimental abdominal aortic aneurysm growth	[169]
*PVT1*	Antisense oligonucleotides	*A*therosclerotic ApoE-deficient mouse model	The expression of LncRNA *PVT1* in the si-LncRNA PVT1 group was reduced. The aortic arch of this group showed a decreased number and size of atherosclerotic plaque compared with the control group, by 70%.	[185]
MicroRNA-19a-3p	Antisense oligonucleotides	HUVEC cell line; atherosclerotic ApoE-deficient mouse model	MiR-19a-3p defines an important regulatory mechanism of endothelial function and the pathogenesis of atherosclerosis.	[198]
Liver-expressed LXR-induced sequence (LeXis)	AAV8 expressing LeXis under the control of the human liver-specific thyroxine-binding globulin promoter	Ldlr-deficient mouse model	*LDLR*−/− animal treatment with AAV8.hTBG.LeXis demonstrated a reduction of almost 2 times in AS load compared to mice in the control group.	[199]
